# Needle-track metastasis in diffuse intrinsic pontine glioma: Need for a standardized surgical strategy?

**DOI:** 10.1093/noajnl/vdag155

**Published:** 2026-06-08

**Authors:** Gunther Nussbaumer, Olga Nigro, Anke Barnbrock, Martin Benesch, Veronica Biassoni, Hans Christoph Bock, Andrea Carai, Anna Cavallo, Silvia Chiesa, Tommaso Giandini, Giovanna Stefania Colafati, Lucia De Martino, Martin Demmert, Valentina di Ruscio, Jeanne-Marie Franke, Lea L Friker, Gerrit H Gielen, Marion Hoffmann, Karolina Jablonska, Axel Karow, Michael Karremann, Kornelius Kerl, Friederike Knerlich-Lukoschus, Christof M Kramm, Mechthild Krause, Sebastian Makocki, Angela Mastronuzzi, Maura Massimino, Andrés Morales La Madrid, Emilia Pecori, Thomas Perwein, Torsten Pietsch, Dirk Rades, Claus Rödel, Vicente Santa-Maria Lopez, Elisabetta Schiavello, Rudolph Schwarz, Sergiu Scobioala, Thorsten Simon, Dorothee C Spille, Ulrich-Wilhelm Thomale, Arianna Trovò, Sabina Vennarini, André O von Bueren, Brigitte Bison, Chiara Valentini

**Affiliations:** Division of Pediatric Hematology and Oncology, Department of Pediatrics and Adolescent Medicine, Medical University of Graz, Graz, Austria; Pediatric Oncology Unit, Fondazione IRCCS Istituto Nazionale dei Tumori, Milano, Italy; Department of Pediatrics, Goethe-University Frankfurt, Frankfurt, Germany; Division of Pediatric Hematology and Oncology, Department of Pediatrics and Adolescent Medicine, Medical University of Graz, Graz, Austria; Pediatric Oncology Unit, Fondazione IRCCS Istituto Nazionale dei Tumori, Milano, Italy; Division of Pediatric Neurosurgery, Department of Neurosurgery, University Medical Center Göttingen, Goettingen, Germany; Neurosurgery Unit, Bambino Gesù Children’s Hospital (IRCCS), Rome, Italy; S.C. Fisica Sanitaria, Medical Physics Unit, Department of Diagnostic Imaging and Radiotherapy, Fondazione IRCCS Istituto Nazionale dei Tumori, Milan, Italy; UOC di Radioterapia Oncologica, Dipartimento Diagnostica per Immagini, Radioterapia Oncologica ed Ematologia, Fondazione Policlinico Universitario Gemelli IRCCS, Rome, Italy; S.C. Fisica Sanitaria, Medical Physics Unit, Department of Diagnostic Imaging and Radiotherapy, Fondazione IRCCS Istituto Nazionale dei Tumori, Milan, Italy; Oncological Neuroradiology and Advanced Diagnostics Unit, Bambino Gesù Children’s Hospital (IRCCS), Rome, Italy; U.O.S.D. Neurooncologia, Ospedale Santobono—A.O.R.N. Santobono-Pausilipon, Napoli, Italy; Department of Pediatric and Adolescent Medicine, Pediatric Hematology and Oncology, University Hospital Schleswig Holstein, Lübeck, Germany; Neuro-Oncology Unit, Hematology/Oncology, Cell Therapy, Gene Therapies and Hematopoietic Transplant, IRCCS Bambino Gesù Children’s Hospital, Rome, Italy; Division of Pediatric Hematology and Oncology, University Medical Center Göttingen, Göttingen, Germany; Institute of Neuropathology, DGNN Brain Tumor Reference Center, University of Bonn Medical Center, Bonn, Germany; Institute of Experimental Oncology, University Hospital Bonn, Bonn, Germany; Brain Tumor Translational Research Group, University Hospital Bonn, Bonn, Germany; Institute of Neuropathology, DGNN Brain Tumor Reference Center, University of Bonn Medical Center, Bonn, Germany; Division of Pediatric Hematology and Oncology, University Medical Center Göttingen, Göttingen, Germany; Department of Radiation Oncology, CyberKnife and Radiation Therapy, Centre for Integrated Oncology Aachen Bonn Cologne Duesseldorf (CIO ABCD), Faculty of Medicine and University Hospital of Cologne, University of Cologne, Cologne, Germany; Pediatric Oncology and Hematology, Department of Pediatrics and Adolescent Medicine, University Hospital Erlangen, Erlangen, Germany; Department of Pediatric and Adolescent Medicine, University Medical Center Mannheim, Medical Faculty Mannheim, Heidelberg University, Mannheim, Germany; Department of Pediatric Hematology and Oncology, University Children’s Hospital Muenster, Muenster, Germany; Division of Pediatric Neurosurgery, Department of Neurosurgery, University Medical Center Göttingen, Goettingen, Germany; Division of Pediatric Hematology and Oncology, University Medical Center Göttingen, Göttingen, Germany; Department of Radiotherapy and Radiation Oncology, Faculty of Medicine, University Hospital Carl Gustav Carus, Technical University Dresden, Dresden, Germany; Partner Site Dresden and German Cancer Research Center (DKFZ), German Cancer Consortium (DKTK), Heidelberg, Germany; OncoRay—National Center for Radiation Research in Oncology, Faculty of Medicine and University Hospital Carl Gustav Carus, Technical University Dresden, Helmholtz-Centre, Dresden, Germany; Helmholtz-Zentrum Dresden—Rossendorf (HZDR), Dresden, Germany; National Center for Tumor Diseases (NCT), Partner Site Dresden, Dresden, Germany; University Hospital and Faculty of Medicine C.G. Carus Dresden, Dresden, Germany; DKFZ Heidelberg, Heidelberg, Germany; Department of Radiotherapy and Radiation Oncology, Faculty of Medicine, University Hospital Carl Gustav Carus, Technical University Dresden, Dresden, Germany; Neuro-Oncology Unit, Hematology/Oncology, Cell Therapy, Gene Therapies and Hematopoietic Transplant, IRCCS Bambino Gesù Children’s Hospital, Rome, Italy; Department of Life Sciences and Public Health, Università Cattolica del Sacro Cuore, Rome, Italy; Pediatric Oncology Unit, Fondazione IRCCS Istituto Nazionale dei Tumori, Milano, Italy; Pediatric Neuro-Oncology Unit, Pediatric Cancer Center Barcelona, Hospital Sant Joan de Deu, Barcelona, Spain; Pediatric Radiotherapy Unit, Fondazione IRCCS Istituto Nazionale dei Tumori, Milan, Italy; Division of Pediatric Hematology and Oncology, Department of Pediatrics and Adolescent Medicine, Medical University of Graz, Graz, Austria; Institute of Neuropathology, DGNN Brain Tumor Reference Center, University of Bonn Medical Center, Bonn, Germany; Department of Radiation Oncology, University of Luebeck, Luebeck, Germany; Department of Radiotherapy, University of Frankfurt, Frankfurt, Germany; Pediatric Neuro-Oncology Unit, Pediatric Cancer Center Barcelona, Hospital Sant Joan de Deu, Barcelona, Spain; Pediatric Oncology Unit, Fondazione IRCCS Istituto Nazionale dei Tumori, Milano, Italy; Department for Radiotherapy, University Medical Center Hamburg-Eppendorf, Hamburg, Germany; Department of Radiation Oncology, Münster University Hospital, Münster, Germany; Department of Pediatric Hematology and Oncology, University of Cologne, Cologne, Germany; Department of Neurosurgery, University Hospital Münster, Münster, Germany; Pediatric Neurosurgery, Charité-Universitätsmedizin Berlin, Berlin, Germany; Pediatric Radiotherapy Unit, Fondazione IRCCS Istituto Nazionale dei Tumori, Milan, Italy; Pediatric Radiotherapy Unit, Fondazione IRCCS Istituto Nazionale dei Tumori, Milan, Italy; Department of Pediatrics, Gynecology and Obstetrics, Division of Pediatric Hematology and Oncology, University Hospital of Geneva, Geneva, Switzerland; Cansearch Research Platform for Pediatric Oncology and Hematology, Faculty of Medicine, Department of Pediatrics, Gynecology and Obstetrics, University of Geneva, Geneva, Switzerland; Diagnostic and Interventional Neuroradiology, Faculty of Medicine, University of Augsburg, Augsburg, Germany; Neuroradiological Reference Center for the Pediatric Brain Tumor (HIT), Studies of the German Society of Pediatric Oncology and Hematology, University Hospital Augsburg, Augsburg, Germany; Department of Radiotherapy and Radiation Oncology, Faculty of Medicine, University Hospital Carl Gustav Carus, Technical University Dresden, Dresden, Germany; Helmholtz-Zentrum Dresden—Rossendorf (HZDR), Dresden, Germany; National Center for Tumor Diseases (NCT), Partner Site Dresden, Dresden, Germany; University Hospital and Faculty of Medicine C.G. Carus Dresden, Dresden, Germany; DKFZ Heidelberg, Heidelberg, Germany

**Keywords:** biopsy track metastasis, diffuse intrinsic pontine glioma, diffuse midline glioma, DMG K27M, H3 K27-altered, radiotherapy

## Abstract

**Background:**

Diffuse intrinsic pontine glioma (DIPG) remains uniformly lethal. Stereotactic biopsy confirms the diagnosis and enables molecular profiling. Metastasis along the biopsy track (BTM) has been reported only anecdotally; its prevalence, clinical relevance, and implications for treatment remain unclear.

**Methods:**

A multicenter retrospective study in patients with confirmed DIPG and BTM was conducted based on central neuroradiologic review. Radiotherapy schedules were re-assessed to evaluate the feasibility of upfront biopsy track irradiation.

**Results:**

Ten children met inclusion criteria (median age 6.8 years). Biopsy route was supratentorial in six and infratentorial in four children, and side-cutting needles were used predominantly. *H3F3A* mutations were most frequent (*n* = 8); *TP53* alterations were common in tumors with extended molecular profiling available. Median PFS was 8.1 months. Five patients each developed BTM prior to (median 2.7 months) or concurrently with progression of primary tumor. There was no difference in overall survival (median OS 12.0 months) compared with the reference cohort. Estimated BTM prevalence among biopsied DIPG from additional registry data was between 6.9% and 13.0%. Primary biopsy track irradiation proved to be feasible, and comparing the surgical access routes, the infratentorial biopsy track hardly increased radiation exposure of the whole brain.

**Conclusions:**

Needle track metastasis is a rare progression pattern in stereotactic biopsied DIPG. Upfront irradiation of the biopsy track may represent a strategy to mitigate the potential risk of BTM. From a dosimetric perspective, an infratentorial approach may therefore be considered, as it was associated with only marginally increased radiation exposure.

Key Points

- Needle track metastasis occurs in approximately 5-10% of biopsied diffuse intrinsic pontine glioma.
- Including the biopsy track in upfront irradiation strategy is feasible.
- To reduce radiation exposure, the infratentorial biopsy access may be considered.

Importance of the Study

Biopsy track metastases (BTM) in diffuse intrinsic pontine glioma represent a rare but clinically relevant procedure-related pattern of progression following diagnostic tumor sampling. While stereotactic biopsy has become increasingly important for molecular characterization and trial eligibility, the incidence, risk factors, and clinical consequences of BTM remain poorly defined in the existing literature. This study provides a systematic characterization of BTM in an international cohort addressing an under-recognized complication with direct implications for clinical practice. Recognition of BTM may inform operative strategies aimed at minimizing needle-track seeding, guide radiotherapy planning by considering coverage of the biopsy trajectory, and improve counseling of families regarding procedure-related risks. These findings provide a foundation for refining surgical and radiotherapeutic approaches in future prospective studies.

Diffuse midline gliomas, H3 K27-altered (DMG), are lethal pediatric tumors arising in eloquent midline structures of the brain, particularly the pons where they are referred to as diffuse intrinsic pontine gliomas (DIPG). Resection is not possible due to tumor localization and infiltrative growth. Standard treatment for DIPG consists of radiotherapy, which provides only transient benefit resulting in a median survival of about 9-12 months, with no curative treatment available.^1-3^

In recent years, the paradigm of tumor sampling in DIPG has changed: histopathologic evaluation had been considered inconsequential since grading was not predictive of survival and, therefore, diagnostic surgery had been abandoned.^4^ A decade ago, driver mutations in histone-3 were established as pathognomonic in DMG.^5^^,^^6^ The presence of these alterations is not only of prognostic significance, but their discovery paved the way for molecular investigations in DIPG. Broad molecular analyses of tumor tissue enable identification of genetic alterations that may be amenable to targeted therapies.^7-10^ More recently, there are several sophisticated efforts that demonstrate feasibility of a prospective, broad molecular assessment in DIPG at diagnosis, and tissue collection through biopsy is currently the only way to conduct this comprehensive molecular workup.^11-13^ Multiple studies have confirmed that stereotactic biopsy of brainstem tumors in children can be performed safely through various techniques with most surgery-associated symptoms being temporary.^13-19^

By contrast, DIPG can be diagnosed with high accuracy based on characteristic radiomorphological features only.^20^^,^^21^ Moreover, clinically actionable genetic alterations suitable for conventional targeted therapies are rare in DMG, which keeps the debate on the rationale for routine biopsy ongoing.

Here we report on metastasis along the biopsy track after stereotactic biopsy in DIPG. This progression pattern has only been reported anecdotally,^22^^,^^23^ however, its prevalence and impact on prognosis have not been elucidated so far. The aim of the present study was to characterize children with DIPG who developed biopsy track metastasis (BTM) as well as to evaluate its clinical implications including surgical procedures and irradiation strategy. Finally, we reflect on the broader implications of our findings for stereotactic biopsy in DIPG in general.

## Methods

### Study Design

Following institutional review board approval by the Medical University of Graz, Austria (IRB number: 36-049 ex 23/24), data collection was conducted in the context of an international multicenter retrospective study including centers in Spain (*n* = 2), Italy (*n* = 3), and Germany (*n* = 5). Inclusion criteria were as follows: (i) presence of a histone-3 alteration; (ii) central neuroradiological confirmation of (a) a tumor with pontine epicenter and diffuse growth pattern compatible with DIPG, and (b) metastasis along the stereotactic needle track; (iii) age at diagnosis ≤18 years. The two Spanish patients reported here had already been published by Lobon-Iglesias et al.^23^ During screening, two patients with non-pontine DMG and BTM following stereotactic biopsy as well as one child with DMG and metastasis along the former track of a ventricular drainage were identified but were not included in the study cohort (for more details see Supplementary Figure S1).

### Data Collection

Clinical data, including basic parameters (e.g. sex, age at diagnosis, overall survival), surgical procedures, and oncological treatment were collected. Confirmation of histone-3 mutations through sequencing and/or immunohistochemistry was mandatory. Apart from histone mutations, additional molecular data on tumor tissue were extracted from preexisting reports if available.

### Neuroradiological Assessment

Central neuroradiologic review of the primary tumor at diagnosis and at onset of metastasis along the stereotactic needle track was mandatory and performed by an experienced pediatric neurooncological radiologist (B.B.). The metastases were confirmed radiologically by the presence of a discrete solid mass along in the former biopsy route, with signal characteristics similar to the primary tumor but without an apparent connection to it. Local progression of DIPG was defined as an increase in tumor volume of more than 25% compared to the previous MRI imaging.

### Irradiation Data and Comparison of Radiotherapy Schedules

To evaluate the different irradiation strategies, an additional IRB approval (BO-EK-118032024) was obtained from the Technical University Dresden. Irradiation plans were available in seven patients. For the purpose of this study, irradiation schedules based on postoperative imaging were generated (RayStation 4.7, RaySearch Laboratories AB, Stockholm, Sweden; Eclipse treatment planning system, version 16.1. Varian Medical Systems, Palo Alto (CA): Varian Medical Systems; 2023) and evaluated by two experienced neuro-oncological radiotherapists (S.V. and C.V.). The prescribed dose was 36 Gy to the biopsy track and 54 Gy to the primary tumor with daily fractions of 1.8 Gy. To define the target volumes, the planning CTs were fused with diagnostic imaging. The gross tumor volume (GTV) was delineated using diagnostic MRI sequences, including T1 with contrast medium, T2, and FLAIR. Post-biopsy MRI scans were used to delineate the biopsy track. For each patient, the primary tumor and biopsy track were delineated: a 1-cm margin was applied to create the clinical target volume (CTV), while the biopsy track itself was considered the CTV. An additional 0.3-cm margin was added to generate the planning target volume (PTV). All cases were planned for photon radiotherapy, and five patients were additionally calculated for proton therapy; in the latter, no margin from CTV to PTV was applied due to robust planning. Treatment planning was performed using commercial treatment planning systems within the Varian ARIA environment and RayStation (RaySearch Laboratories). VMAT plans were generated using inverse planning, in which the treatment system optimizes the beam delivery to achieve the prescribed dose. The calculation accounted for differences in tissue density. A dose grid of 2-3 mm was used depending on the target size and anatomy. Proton treatment planning was performed in RayStation (RaySearch Laboratories) for pencil beam scanning delivery. Dose calculation accounted for tissue heterogeneities, with dose reported as absorbed dose to water in Gy (RBE), assuming a constant relative biological effectiveness of 1.1. Robust optimization was applied to account for setup and range uncertainties, and a dose calculation grid of 2-3 mm was used depending on target size and anatomical complexity. Target coverage and organ-at-risk constraints were evaluated using dose-volume histogram metrics, with volumetric dose reporting (D98%, D50%, D2%) performed in accordance with “International Commission on Radiation Units and Measurements” (ICRU) recommendations. The major organs at risk considered were the brain, the optic chiasm, and the brainstem. For photon plans, the entire brain excluding the PTV was contoured, and dose constraints were evaluated for this healthy brain volume. For proton plans, robust optimization was performed without defining a PTV, and the volume of healthy brain tissue receiving radiation was evaluated in cubic centimeters.

### Statistical Analyses and Reference Cohort

Data analysis was carried out using R Core Team 2020 (v4.4.1)^24^ using the packages “survminer” (v0.4.9)^25^ and “survival’”(v3.6-4).^26^ Survival analyses were performed using the log-rank test, and Kaplan-Meier estimates. Comparisons of qualitative parameters between two groups were conducted using Fisher’s exact test. A two-sided alpha level of 0.05 was used as the threshold for statistical significance.

**Figure 1.**
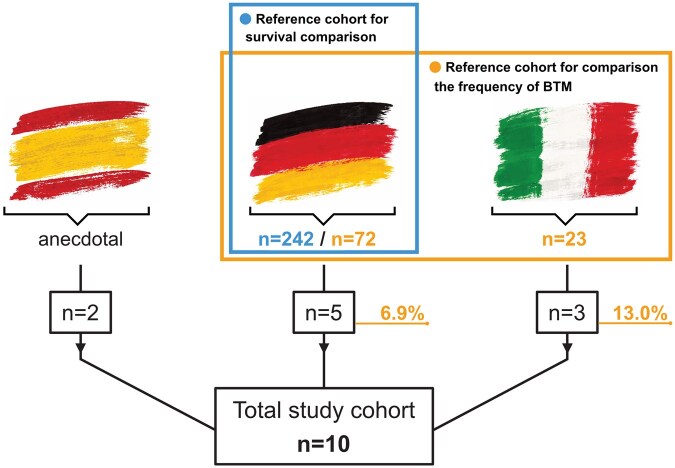
Flowchart illustrating patient numbers and the different reference cohorts for the various outcome parameters.

As a reference cohort for survival comparison, all confirmed DIPG patients (*n* = 242) from the prospective German pedHGG studies HIT-HGG-2007 (Eudra-CT 2007-010128-42; *n* = 162) and HIT-HGG-2013 (EudraCT 2013-004187-56; *n* = 80) were assembled.^27^ To estimate the relative frequency of BTM in DIPG, all DIPG patients who underwent stereotactic biopsy at diagnosis were identified both in the above-mentioned German (*n* = 72 of 242 enrolled patients) and Italian (NCT03620032; *n* = 23 of 55) reference cohorts. Both trials include children and adolescents only. The study flow chart of patient recruitment and the different reference cohorts is shown in Figure 1.

## Results

### Characteristics of the Study Cohort

Through this international multicenter effort, a total of 10 patients with BTM in DIPG were identified. A comprehensive compilation of essential clinical features is given in Table 1 and Supplementary Table S1. Based on available staging at diagnosis, there was no evidence of disseminated disease in any of these patients. Median age at diagnosis was 6.8 years (range: 3.7-12.9; equal sex distribution). Eight patients underwent biopsy within less than 6 weeks after symptom onset. In all patients, a stereotactic surgical approach was chosen. Frame-based techniques as well as frameless procedures including robotic assistance in three surgeries were performed with equal frequency. The biopsy track was supratentorial in six and infratentorial in four patients. The latter were more frequently biopsied using frameless techniques (in 3/4 cases). In all cases with available data (8/10), side-cutting needles were used for biopsy sampling in seven patients and a biopsy forceps in one patient.

**Table 1.** Comprehensive clinical characteristics of the whole cohort

Basic characteristics			*n* = 10
Sex		Male: female	5:5
Age at diagnosis (in years)		Median	6.8
		Range	3.7-12.9
Duration of symptoms		<6 Weeks	8
		6-12 Weeks	2
**Diagnostic data**			***n* = 10**
Staging	Spinal MRI at diagnosis	Performed	6
		Disseminated disease	0
		Not performed	2
		Not available	2
	CSF evaluation at diagnosis	Performed	3
		Malignant cells	0
		Not performed	4
		Not available	3
Surgical data	Stereotaxy	Frame-based	5
		Frameless	5
		Robot-assisted	2
	Biopsy tool	Side cutting needle	7
		Forceps needle	1
		Unknown	2
	Surgical access	Supratentorial	6
		Infratentorial	4
	Histone mutation	*H3F3A*	8
		*HIST1H3B*	1
		*HIST2H3C*	1
**Treatment data**			***n* = 10**
Irradiation			10
Adjuvant treatment			10
Temozolomide			5
Nimotuzumab + vinorelbine			3
MEMMAT-like^a^			1
Ribociclib + everolimus			1
Re-irradiation^b^			7
**Progression and survival data**			***n* = 10**
Progression pattern			10
Biopsy tract metastasis first			5
Local progression and BTM simultaneously			5
Time between BTM and local progression (in months)		Median	2.7
		Interquartile range	1.5-4.4
Time-to-local progression (in months)		Median	8.1
		Interquartile range	6.1-9.2
Survival status		Dead	10
Overall survival (in months)		Median	12.0
		Interquartile range	10.1-16.7

a Agents used: etoposide, celecoxib, fenofibrate, thalidomide;

b Re-irradiation included biopsy track metastasis; abbreviations: cerebrospinal fluid (CSF), magnetic resonance imaging (MRI).

Histone-3 mutation was confirmed by sequencing in all tumors, with *H3F3A* (*n* = 8) being the most prevalent; *HIST1H3B* and *HIST2H3C* mutations were detected in one tumor each. TP53 mutations were present in four of five tumors with available exome sequencing data.

**Figure 2.**
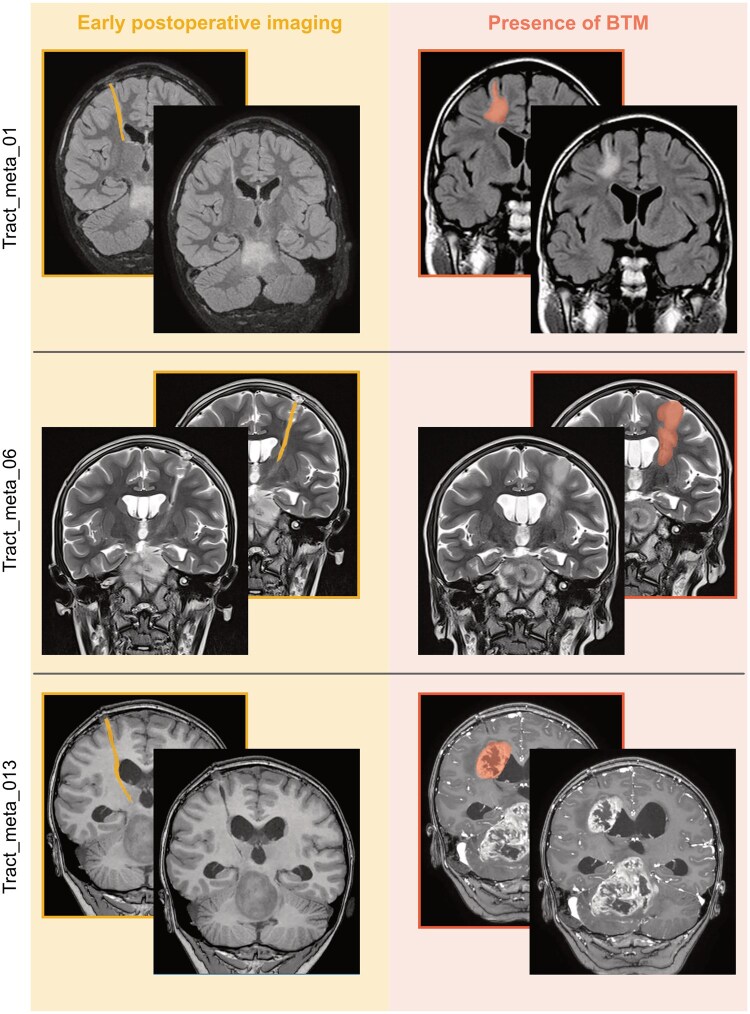
Representative examples of biopsy track metastasis (BTM). Postoperative magnetic resonance images are shown on the left (yellow) with the biopsy trajectory, and follow-up images after emergence of BTM are shown on the right (red). Color-overlay duplicates are provided to facilitate visualization of the biopsy trajectory and needle-track metastasis.

Following biopsy, all patients received local irradiation and adjuvant chemotherapy, most commonly temozolomide (TMZ). All children experienced local progression: in half of the patients local progression and BTM occurred simultaneously; the other five patients developed BTM prior to progression of the primary tumor with a median interval of 2.7 months (range 1.5-4.4 months). Median progression-free survival (PFS, either local progression or presence of BTM) was 8.1 months (interquartile range [IQR]: 6.1-9.2). Representative examples of BTM are presented in Figure 2. Moreover, five patients additionally experienced leptomeningeal dissemination at progression. Seven children received re-irradiation at progression, and re-irradiation strategy (craniospinal or focal) included biopsy track metastasis.

**Figure 3.**
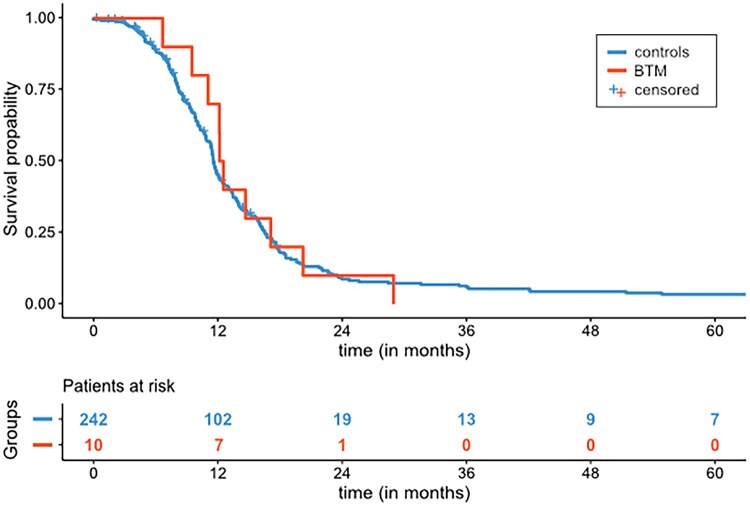
Comparative survival analysis between the BTM study cohort and control group. Kaplan-Meier plot including patients at risk calculation comparing overall survival of BTM study (orange) and children with DIPG (blue) derived from the German pedHGG HIT trials (*n*=242). There was no significant difference in survival between these two groups (*P* = .817).

All children succumbed to their disease with a median overall survival (OS) of 12.2 months (IQR 10.1-16.7). Compared to the control cohort of DIPG patients (*n* = 242) homogeneously treated with radiotherapy and adjuvant TMZ, children with BTM showed no significant difference in survival (median OS 12.2 vs. 11.5 months [IQR 7.9-16.0]; *P *= .817; Figure 3). Interestingly, one child with rare *HIST2H3C*-mutation showed prolonged survival (29 months) and developed BTM more than 20 months after biopsy.

To estimate the relative frequency of BTM in DIPG, all stereotactically biopsied DIPG patients derived from the German (*n* = 72) and Italian (*n* = 23) cohorts, from which the study patients were derived, were used as reference. The estimated frequency for the five German patients was 6.9%, for the three Italian cases 13.0% (Figure 1). In the German control cohort, the biopsy route was supra—in 35/72 (49%) and infratentorial in 37/72 patients (51%). In the Italian cohort, the surgical access was supra- and infratentorial in 19/23 (83%) and 4/23 (17%) children, respectively. Compared with the corresponding control group, BTM was not significantly associated with the supra- or infratentorial surgical access route in either the German (*P* = .358) or the Italian (*P* = .488) cohort.

### Comparison of Different Irradiation Strategies

**Figure 4.**
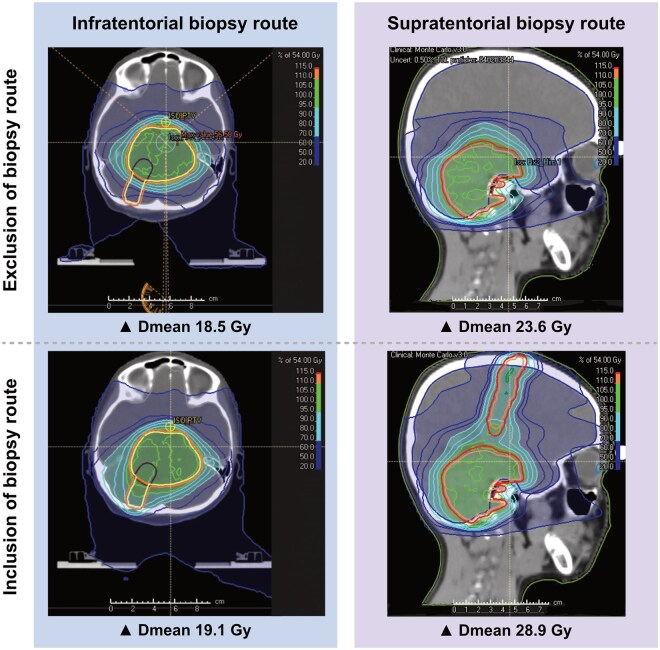
Comparison of photon irradiation strategies according to biopsy approach. A representative treatment plan is shown for an infratentorial biopsy approach on the left and for a supratentorial biopsy approach on the right. The upper row shows radiation exposure without biopsy track inclusion, whereas the lower row shows radiation exposure with biopsy tract inclusion. Mean radiation exposure (*D*_mean_) to the brain outside the CTV is given for each scenario.

In none of the patients, the biopsy track was included in the CTV during primary irradiation. Based on available radiotherapy plans (*n* = 7), radiation delivered to the needle track during first-line treatment reached at most the 50% isodose for infratentorial (27 Gy), and the 20% isodose (10.8 Gy) for supratentorial biopsy approaches. In a second step, the radiotherapy schedules were reevaluated to assess the feasibility of including the needle track into upfront irradiation strategy. Considering the average radiation exposure for the entire brain outside the CTV, in photon irradiation the inclusion of an infratentorial surgical access route resulted in increased radiation exposure of about 1 Gy compared to conventional irradiation strategies (D_mean_ 16.2 vs. 15.6 Gy). In contrast, inclusion of supratentorial biopsy tracks increased additional exposure by approximately 5 Gy (D_mean_ 22.3 vs. 17.1 Gy) (Figure 4). Conducting the same comparison with proton therapy (*n* = 5), particle beam therapy reduced the exposure to the brain in general, and the inclusion of the supratentorial needle track increased the radiation exposure by 3.2 Gy (D_mean_ 17.4 vs. 14.2 Gy) only. No significant differences were observed for other relevant organs at risks analyzed (chiasma and brainstem excluded CTV).

## Discussion

Needle-track metastasis in glioma is a rare phenomenon. There are only few case reports published in adults with high-grade glioma^28-34^; clinical features and prognostic impact of BTM in children with DIPG are not yet established.

Taking into account the limitations imposed by the small sample size, we consider the prevalence of BTM to be between 5 and 10% and, consequently, to represent a rare event. Due to its rarity, the mechanisms underlying BTM remain uncertain, and its pathogenesis remains poorly understood. BTM most likely reflects iatrogenic seeding of tumor cells along the biopsy trajectory. This interpretation is supported by the characteristic localization of BTM along the biopsy track and by experimental evidence showing that viable H3 K27-altered DMG cells can be recovered from biopsy probes after stereotactic sampling.^35^ There is no consensus on the most efficient and safest procedure for performing stereotactic biopsy in DIPG, and no prospective trials comparing different techniques exist. A survey among neurosurgeons identified frame-based (consensus 88%) and frameless stereotaxy (83%) as the most accepted techniques, and side-cutting needles were mentioned preferentially (77%). Both biopsy access routes, transcerebellar and supratentorial, were generally considered suitable, but the infratentorial approach was the preferred approach in the survey (90% vs. 58%).^36^ Given the small sample size, we could not identify any surgery-related parameter associated with BTM in our study. The supratentorial approach was not significantly related to BTM, even though BTM occurred more frequently in the Italian cohort, in which the supratentorial approach was preferred. The supratentorial route is markedly longer, which may increase the risk of tumor cell displacement during retraction of the biopsy device. However, additional important surgical parameters, such as the frequency of retractions or the tumor region targeted, could not be assessed due to the study design.

In addition to iatrogenic seeding, H3 K27M-altered DMG cells themselves possess a high potential to disseminate.^4^^,^^37-40^ Likewise, in half of our patients, leptomeningeal dissemination was detected at progression indicating a high migratory capacity in our tumors. The underlying molecular mechanisms of invasion in DMG are still poorly understood.^40^ Data on the genetic repertoire of our tumors are scant and the relatively high frequency of *TP53* mutations in our cohort could be due to bias related to the limited number of patients. *TP53* alterations in general frequently coincide with H3 K27-altered DIPG, with a prevalence ranging from 40% up to 80%.^6^^,^^41-43^ In a recently published study on migration in H3 K27M-altered DMG, *TP53* was not associated with the propensity to invade.^44^ However, *TP53*-altered DMG show higher resistance to external influences such as irradiation or certain drugs e.g. ONC201.^45-47^ We hypothesize that *TP53* mutations may enhance the resilience of tumor cells and thereby facilitate survival and outgrowth after displacement to other brain regions. Taken together, our findings support iatrogenic seeding along the biopsy trajectory as the most plausible mechanism underlying BTM, while the invasive biology and resilience of DMG cells may facilitate subsequent survival and outgrowth of displaced tumor cells.

Clinical characteristics (age, sex, treatment strategy) were comparable between our cohort and those reported in the literature.^2^ Remarkably, survival of patients developing BTM was similar to that of cohorts of DIPG patients without BTM. As BTM was included in the re-irradiation, the same survival may indicate a treatment effect on metastasis as well. This raises the question of whether the biopsy track should be included upfront in the irradiation strategy. In this context, it is striking that BTM consistently occurred before or at the time of local progression, but never subsequent to local progression. The surgical access route in DIPG per se is not irradiated routinely. Tumor cells within the needle track, especially in supratentorial approaches, remain unaffected by radiotherapy and may proliferate resulting in the development of solid metastases. Even though survival was not impaired, the occurrence of BTM might cause additional symptoms and increase morbidity, potentially affecting quality of life during the remaining disease course. Prospective trials to longitudinally assess quality of life in DIPG patients are therefore urgently needed.

As a strategy to mitigate the potential risk of BTM, we propose considering inclusion of the needle track in upfront irradiation planning as our data showed that this irradiation strategy is feasible. The use of needle-track irradiation does not substantially impact the radiotherapy planning or delivery workflow. Indeed, it does not require modifications to the simulation procedures, nor does it extend the overall treatment time. Supratentorial biopsy approaches frequently require the addition of an extra irradiation field to adequately encompass the biopsy trajectory. This technical aspect may result in a larger volume of otherwise uninvolved brain tissue being unnecessarily exposed to radiation. By contrast, the infratentorial approaches generally allow a more limited and geometrically favorable field arrangement, often avoiding the need for additional field extensions along the biopsy trajectory resulting in only minimal additional whole-brain exposure. Therefore, from a dosimetric perspective, an infratentorial biopsy approach may be considered when surgically safe and technically feasible.

Our results need to be interpreted carefully in the context of DIPG and must not be misinterpreted as an argument against biopsy in patients with DIPG. Firstly, metastasis along the needle track is a rare phenomenon. Secondly, the presence of BTM did not affect survival. Finally, avoiding biopsies prevents individualized treatment plans based on molecular analyses derived from tissue samples. In addition, the obtained genetic data might enable the development of new treatment strategies which will benefit future generations of patients. We are in line with the recommendation of the pediatric neuro-oncology community: children with DIPG should undergo biopsies within prospective clinical trials, not merely to investigate survival or new drugs, but to monitor quality of life and progression patterns such as BTM.^36^^,^^48-50^

In conclusion, our study suggests that BTM in DIPG is rare and may reflect procedure-related tumor cell displacement, potentially facilitated by biological factors promoting tumor cell survival and outgrowth. We were able to demonstrate that upfront irradiation of the biopsy track is feasible and only marginally increases radiation exposure in infratentorial surgical approaches.

## Supplementary Material

vdag155_Supplementary_Data

## Data Availability

Data that support the findings of this study are available from the corresponding author upon reasonable request.
