# Supplementary material for: Needle-track metastasis in diffuse intrinsic pontine glioma: Need for a standardized surgical strategy?
Source: Neurooncol Adv. 2026 Jun 8;8(1):vdag155. doi: 10.1093/noajnl/vdag155 (PMC13302791; doi:10.1093/noajnl/vdag155)
Supplement: vdag155_Supplementary_Data [file vdag155_supplementary_data.zip › Supplementary_Data (4)/Supp-Table1_241028.docx]

| **ID** | **sex/age^1^** | **surgical access** | **biopsy technique** | | **histone alteration** | **additional alterations^2^** | **irradiation dose^3^** | **adjuvant**  **chemotherapy** | **PFS^4^** | **OS^4^** |
| --- | --- | --- | --- | --- | --- | --- | --- | --- | --- | --- |
| Tract_meta_01 | M/8 | supratentorial | frame-based | side-cutting needle | *HIST1H3B* | N/A | 52.2 | TMZ | 9.2 | 20.4 |
| Tract_meta_03 | F/7 | infratentorial | frame-based | side-cutting needle | *H3F3A* | N/A | 54 | TMZ | 8.7 | 12.7 |
| Tract_meta_04 | F/6 | supratentorial | frame-based | N/A | *H3F3A* | N/A | 54 | TMZ | 7.4 | 12.2 |
| Tract_meta_06 | F/7 | supratentorial | frameless;  robot-assisted | Side-cutting needle | *H3F3A* | *TP53, CDK4* | N/A | NIMO+VINO | 10.5 | 17.2 |
| Tract_meta_07 | M/6 | supratentorial | frameless;  robot-assisted | side-cutting needle | *H3F3A* | *TP53* | 59.4 | NIMO+VINO | 5.9 | 14.9 |
| Tract_meta_09 | M/6 | infratentorial | frameless | side-cutting needle | *H3F3A* | N/A | 39 | VP16+VPA+celecoxib  +fenofibrate+THAL | 6.0 | 11.3 |
| Tract_meta_10 | F/6 | infratentorial | frameless | side-cutting needle | *H3F3A* | N/A | 39 | EVE+Ribociclib | 6.5 | 9.9 |
| Tract_meta_12 | F/3 | infratentorial | frameless;  robot-assisted | side-cutting needle | *HIST2H3C* | *ACVR1* | 75.6 | NIMO+VINO | 17.3 | 29.0 |
| Tract_meta_13 | M/9 | supratentorial | frame-based | forceps-needle | *H3F3A* | *TP53, PIK3R1* | 54 | TMZ | 3.5 | 6.9 |
| Tract_meta_14 | M/12 | supratentorial | frame-based | N/A | *H3F3A* | *TP53* | 60 | TMZ | 9.0 | 12.2 |

**Supplementary Table 1: Clinical features by patient**

^1^age at diagnosis in years; ^2^an excerpt of relevant alterations, ^3^irradiation dose in Gray; ^4^in months; abbreviations: female (F), male (M), temozolomide (TMZ), nimotuzumab (NIMO), vinorelbine (VINO), thalidomide (THAL), everolimus (EVE), progression-free survival (PFS) in months, overall survival (OS) in months
