# Supplementary material for: Needle-track metastasis in diffuse intrinsic pontine glioma: Need for a standardized surgical strategy?
Source: Neurooncol Adv. 2026 Jun 8;8(1):vdag155. doi: 10.1093/noajnl/vdag155 (PMC13302791; doi:10.1093/noajnl/vdag155)
Supplement: vdag155_Supplementary_Data [file vdag155_supplementary_data.zip › Supplementary_Data (4)/Supplementary_Figure_Legend_NOA-D-26-00070.docx]

# **Supplementary Figure Legend**

**Supplementary Figure 1: Collage of cases with non-pontine DMG and dissemination through preexisting surgical routes**

The first two patients (*Track_meta_05* and *Track_meta_08*) had thalamic K27M DMG. On the left side in yellow: in the front the primary tumor (doted circled), in the background, the biopsy route (linear circled) is presented. On the right in red, imaging after the emergence of biopsy track metastasis is shown (BTM is marked red in the respective copy). The last patient (*Track_meta_11*) developed hydrocephalus following DMG (doted circled) and, subsequently, metastases along a former ventricular drainage (circled in green). These three cases were not included in the study cohort.
