# Supplementary figures and images for: Needle-track metastasis in diffuse intrinsic pontine glioma: Need for a standardized surgical strategy?
Source: Neurooncol Adv. 2026 Jun 8;8(1):vdag155. doi: 10.1093/noajnl/vdag155 (PMC13302791; doi:10.1093/noajnl/vdag155)

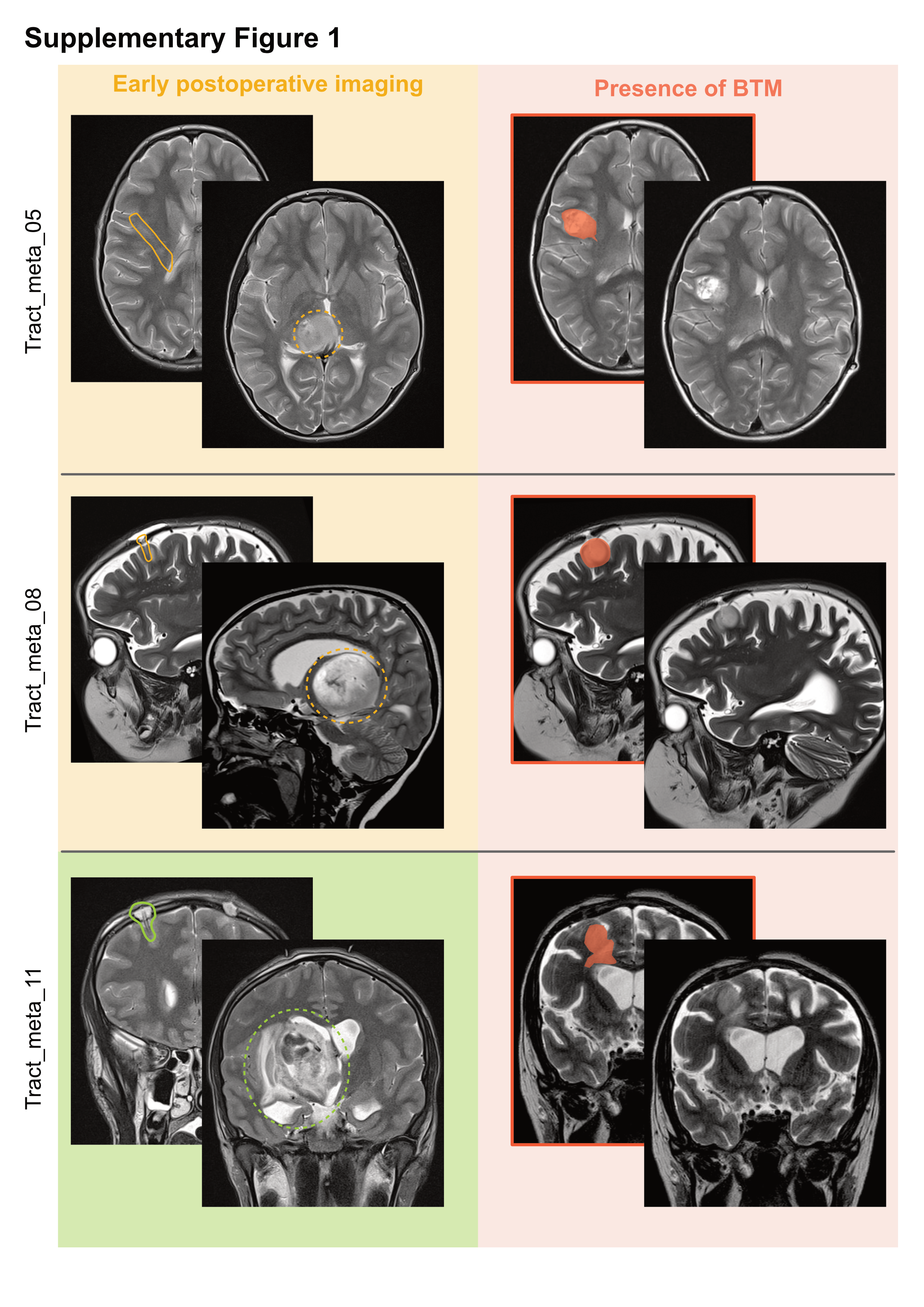

Supplement: vdag155_Supplementary_Data [file vdag155_supplementary_data.zip › Supplementary_Data (4)/Supp.Figure_1.tiff]
